# Identification of a Missing Link in the Evolution of an Enzyme into a Transcriptional Regulator

**DOI:** 10.1371/journal.pone.0057518

**Published:** 2013-03-19

**Authors:** Gonzalo Durante-Rodríguez, José Miguel Mancheño, Germán Rivas, Carlos Alfonso, José Luis García, Eduardo Díaz, Manuel Carmona

**Affiliations:** 1 Department of Environmental Biology, Centro de Investigaciones Biológicas-CSIC, Madrid, Spain,; 2 Group of Crystallography and Structural Biology, Institute of Physical Chemistry Rocasolano, CSIC, Madrid, Spain,; 3 Department of Chemical and Physical Biology, Centro de Investigaciones Biológicas-CSIC, Madrid, Spain; Univeristy of California Riverside, United States of America

## Abstract

The evolution of transcriptional regulators through the recruitment of DNA-binding domains by enzymes is a widely held notion. However, few experimental approaches have directly addressed this hypothesis. Here we report the reconstruction of a plausible pathway for the evolution of an enzyme into a transcriptional regulator. The BzdR protein is the prototype of a subfamily of prokaryotic transcriptional regulators that controls the expression of genes involved in the anaerobic degradation of benzoate. We have shown that BzdR consists of an N-terminal DNA-binding domain connected through a linker to a C-terminal effector-binding domain that shows significant identity to the shikimate kinase (SK). The construction of active synthetic BzdR-like regulators by fusing the DNA-binding domain of BzdR to the *Escherichia coli* SKI protein strongly supports the notion that an ancestral SK domain could have been involved in the evolutionary origin of BzdR. The loss of the enzymatic activity of the ancestral SK domain was essential for it to evolve as a regulatory domain in the current BzdR protein. This work also supports the view that enzymes precede the emergence of the regulatory systems that may control their expression.

## Introduction

Regulation of transcription through the action of small molecules that directly bind to a transcription factor is widespread in all life forms. A large number of transcriptional regulators contain a DNA-binding domain fused to an effector-binding domain. Binding of the effector results in a conformational change, which influences the properties of the transcription factor and, accordingly, results in activation or repression of transcription [1].

The effector-binding protein domains of transcriptional regulators appear to have evolved by distinct selective forces. In some cases, the effector-binding protein domains appear to derive from catalytic proteins, which may or may not retain the active site residues in their binding pockets during evolution and thus could possibly behave as bifunctional proteins [2]. There are also a few examples of transcriptional regulators that might have evolved from enzymes that have lost their catalytic activity. In eukaryotes, we could mention the Gal80 regulator involved in the catabolism of galactose in *Saccharomyces cerevisiae* and *Kluyveromyces lactis* [3], and TAFII150 protein in *Drosophila melanogaster* which controls transcription mediated by the RNA polymerase (RNAP)-II [4]. In prokaryotes, the HutC regulator of *Pseudomonas putida*, involved in histidine utilization [5], and the FarR regulator of *Escherichia coli*, which controls the expression of Krebs cycle genes and responds to fatty acids [6], contain effector-binding domains similar to chorimaste lyases [2]. However, the evolutionary pathways giving rise to these regulators have not yet been reproduced in the laboratory.

Anaerobic pathways provide reliable model scenarios to study protein evolution since they may preserve relics of ancient events occurring on early anaerobic Earth. In an effort to understand the evolution of regulators, we have reconstructed the most probable evolutionary pathway followed by the BzdR regulator. The BzdR protein of the facultative anaerobe β-Proteobacterium *Azoarcus* sp. CIB is the prototype of a new subfamily of transcriptional regulators. This regulator controls the expression of genes involved in the aerobic or anaerobic degradation of benzoate [7].

**Figure 1:**
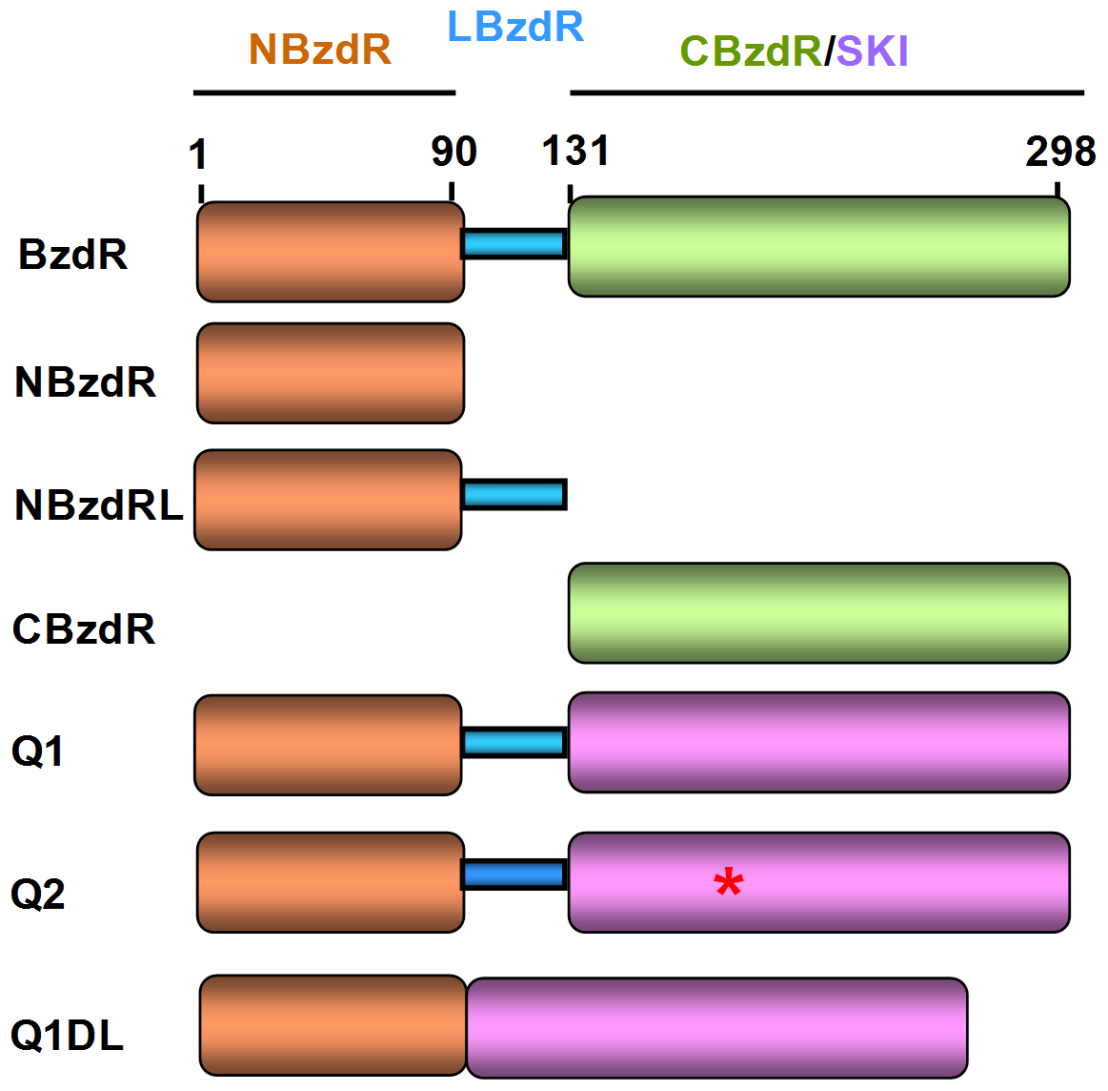
Modular architecture of the BzdR derivatives. Diagram showing the modular architecture of the BzdR protein, its NBzdR, NBzdRL and CBzdR domains, and the Q1, Q2 and Q1ΔL chimeras. The N-terminal domain (NBzdR), C-terminal domain (CBzdR) and linker region of BzdR are indicated in orange, blue and green, respectively. The *E. coli* SKI enzyme is shown in violet, and the Asp168Ala substitution in the Q2 chimera is indicated by an asterisk in red.

The predicted domain organization of BzdR consists of an N-terminal region (NBzdR, residues 1–90), homologous to the DNA-binding domain of members of the helix-turn-helix (HTH)-XRE transcriptional regulator, connected through a linker sequence to a C-terminal region (CBzdR, residues 131–298), This C-terminal region shows 23% sequence identity with the *E. coli* shikimate kinase I (SKI) (aroK gene product). In addition CBzdR conserves the SKI P-loop-containing nucleoside triphosphate hydrolase fold, the Walker-A motif and the Gly present in the Walker B-motif of purine nucleotide-binding proteins, and recognizes the inducer molecule benzoyl-CoA (Figure 1) [7], [8]. In this work, we have experimentally reproduced the most likely evolutionary pathway followed by the BzdR protein. First, we show that NBzdR and CBzdR are real functional domains able to bind DNA and the effector molecule, respectively. We then constructed functionally active synthetic BzdR-like regulators by fusing the DNA-binding domain of BzdR to *E. coli* SKI protein as the effector-binding domain. The observed functionality of these synthetic regulators provides strong support for a role of an ancestral SK enzyme in the evolutionary origin of the BzdR protein.

## Materials and Methods

### Strains, Plasmids, Growth Conditions, and Molecular Biology Procedures

The *E. coli* strains and plasmids used in this work are listed in Table 1. The construction of recombinant plasmids featuring the BzdR modules and the chimeras Q1, Q1ΔL and Q2 is detailed in the supplementary materials. *E. coli* cells were grown on Luria-Bertani (LB) [9] or M63 [10] medium supplemented with the appropriate carbon source at 37°C. Anaerobic growth in LB medium using 10 mM nitrate as the final electron acceptor was achieved as previously reported [7]. Standard molecular biology techniques were performed as described previously [9].

**Table 1:** Bacterial strains and plasmids used in this work.

Strain or plasmid	Relevant phenotype and/or genotype^a^	Reference
*E. coli* strains		
DH5α	*endA1 hsdR17 supE44 thi-1 recA1 gyrA*(Nal^r^) *relA1Δ(argF-lac)* ø*80dlacΔ(lacZ)M15*	[9]
M15	Strain for regulated high level expression with pQE vectors	Qiagen
MC4100	F^−^, *araD319* Δ(*argF-lac*)*U169 rpsL150*(Sm^r^) *relA1 flbB5301 deoC1 ptsF25* *rbs*	[29]
AFMC	MC4100 spontaneous Rf^r^	[30]
AFMCP_N_	Rf^r^, Km^r^, AFMC harbouring *P_N_::lacZ* translational chromosomal fusion	[31]
ALO807	F^−^, *aroL478:Tn10 aroK17:Cm^r^*	[18]
CC118	Rf^r^, Sp^r^, Δ(*ara-leu*) *araD*, *ΔlacX74*, *galE*, *galK*, *thi-1*, *rpsE*, *rpoB*, *argE* (Am), *recA1*	[32]
Plasmids		
pQE32	Ap^r^, *ori*ColE1, T*5* promoter *lac* operator, λ t_o_/*E. coli rrnB T1* terminators	Qiagen
pQE32-His_6_BzdR	Ap^r^, pQE32 derivative harboring the His_6_-*bzdR* gene	[7]
pQE32-His_6_NBzdR	Ap^r^, pQE32 derivative harboring the His_6_-*NbzdR* fragment	This work
pQE32-His_6_NBzdRL	Ap^r^, pQE32 derivative harboring the His_6_-*NbzdRL* fragment	This work
pQE32-His_6_CBzdR	Ap^r^, pQE32 derivative harboring the His_6_-*CbzdR* fragment	This work
pQE32-His_6_Q1	Ap^r^, pQE32 derivative harboring the His_6_-*Q1* gene	This work
pQE32-His_6_Q2	Ap^r^, pQE32 derivative harboring the His_6_-*Q2* gene	This work
pQE32-His_6_Q1ΔL	Ap^r^, pQE32 derivative harboring the His_6_-*Q1ΔL* gene	This work
pQE32-His_6_SKI	Ap^r^, pQE32 derivative harboring the His_6_-*aroK* gene	This work
pJCD-P_N_	Ap^r^, pJCD01 derivative harboring a 585-bp *Eco*RI	[12]
pQE60-His_6_-FNR*	Ap^r^, pQE60 derivative that harbors the His_6_-fnr* gene	[12]
pREP4	Km^r^, plasmid that expresses the *lacI* repressor	Qiagen
pECOR7	Ap^r^, pUC19 harboring a 7.1-kb DNA fragment containing the *bzdRNO* genes	[31]
pGEM-Teasy	Ap^r^, *ori*ColE1, *lacZα*, vector for cloning PCR products	Promega
pGEMT-NBzdRL	Ap^r^, pGEM-Teasy derivative harboring the *NbzdRL* fragment	This work
pGEMT-NBzdR	Ap^r^, pGEM-Teasy derivative harboring the *NbzdR* fragment	This work
pGEMT-CBzdR	Ap^r^, pGEM-Teasy derivative harboring the *CbzdR* fragment	This work
pGEMT-SKI	Ap^r^, pGEM-Teasy derivative harboring the *aroK* fragment	This work
pGEMT-Q1	Ap^r^, pGEM-Teasy derivative harboring the *Q1* fragment	This work
pCK01	Cm^r^, *ori*pSC101, low copy number cloning vector, polylinker flanked by *Not*I sites	[33]
pCK01BzdR	Cm^r^, pCK01 derivative harboring a DNA fragment containing the *bzdR* gene	[7]
pCK01Q1	Cm^r^, pCK01 derivative harboring the *Q1* gene	This work
pCK01Q2	Cm^r^, pCK01 derivative harboring the *Q2* gene	This work
pCK01NBzdR	Cm^r^, pCK01 derivative harboring the *NBzdR* gene	This work
pCK01NBzdRL	Cm^r^, pCK01 derivative harboring the *NBzdRL* gene	This work
pSJ3P_N_	Ap^r^, pSJ3 derivative harbouring the translational fusion *P_N_::lacZ*	[7]

Ap^r^, ampicillin resistant; Cm^r^ chloramphenicol resistant; Km^r^, kanamycin resistant; Nal^r^, nalidixic acid; Rf^r^, rifampicin resistant; Sm^r^, streptomycin resistant; Sp^r^, spectinomycin resistant.

### Enzymatic Assays

β-Galactosidase activities were measured using permeabilized cells as described by Miller [10]. Shikimate kinase assays were performed according to previously established procedures [11] with the modifications detailed below. Plasmid pJCD-P_N_ was used as as supercoiled *P_N_* template for *in vitro* transcription assays conducted as previously described [12].

### Gel Retardation and DNase I Footprinting Assays

The *P_N_* DNA probe was obtained and mixed with the purified proteins at the concentrations indicated in each assay according to a previously described procedure [7].

### Recombinant Plasmid Constructions

To clone the NBzdR, NBzdRL and CBzdR domains, *NbzdR*, *NBzdRL* and *CbzdR* fragments were first PCR amplified from pECOR7 plasmid using the oligonucleotide pairs 5′HisReg (5′-CGGGATCCTTTCCAACGATGAGAACTCATCAC-3′; engineered *Bam*HI site underlined)/N1BzdR (5′-GGGAAGCTTTCACTCCGCCTCCTCGCGCACG-3′; engineered *Hin*dIII site underlined), 5′HisReg (see above)/N2BzdR (5′-GGGAAGCTTTCACCTGCGCGCGCTTCGCCCC-3′; engineered *Hin*dIII site underlined) and 5′C2 (5′-CGGGATCCTCAGCGTGCCAGGACTTCGAGG-3′; an engineered *Bam*HI is underlined)/3′HisReg (5′-GGGAAGCTTTCAGCGTGCCAGGACTTCGAGG-3′; engineered *Hin*dIII site underlined). Next, the PCR amplified products were cloned into the pQE32 vector by *Bam*HI/*Hin*dIII double digestion, giving rise to plasmids pQE32-His_6_NBzdR, pQE32-His_6_NBzdRL and pQE32-His_6_CBzdR, respectively. The *NbzdR* and *NBzdRL* fragments were also subcloned under the control of the *Plac* promoter into the pKC01 vector by *Eco*RI/*Hin*dIII double digestionyielding plasmids pCK01NBzdR and pCK01NBzdRL, respectively. The *aroK* gene that encodes the SKI enzyme was PCR amplified from the *E. coli* CC118 genome using 5′His-aroK oligonucleotides (5′-CGGGATCCTTGCAGAGAAACGCAATATCTTT-3′; engineered *Bam*HI site underlined)/5′aroKquim (5′-AACTGCAGTTAGTTGCTTTCCAGCATGTGAATA-3′; engineered *Pst*I underlined) and then cloned into the pQE32 vector by *Bam*HI/*Pst*I double digestion, giving rise to plasmid pQE32- His_6_SKI.

To construct the Q1 and Q1ΔL chimeras, *NBzdR*, *NBzdRL* and *aroK* were first PCR amplified from pECOR7 plasmid and *aroK* from the *E. coli* CC118 genome using the oligonucleotide pairs 5′HisReg (see above)/newN1quim (5′-GGGGTACCCTCCGCCTCCTCGCGCACG-3′; engineered *Kpn*I site underlined) or 5′HisReg (see above)/5′N2qim (5′-GGGGTACCCCTGCGCGCGCTTCG-3`; engineered *Kpn*I site underlined) and 3′aroKqim* (5′-GGGGTACCATGGCAGAGAAACGCAATATCTTT-3′; engineered *Kpn*I site underlined)/5′aroKquim (see above), respectively. The NBzdR and NBzdRL amplified products were cloned into the pGEM-Teasy vector by *Bam*HI/*Kpn*I double digestion, giving rise to plasmids pGEMT-NBzdR and pGEMT-NBzdRL, respectively. The *aroK* amplified product was then cloned into the pGEMT-NBzdR and pGEMT-NBzdRL vectors by *Kpn*I/*Pst*I double digestion, giving rise to plasmids pGEMT-Q1ΔL and pGEMT-Q1. These constructions were digested using *Bam*HI and *Pst*I enzymes to subclone the Q1ΔL and Q1 genes with N-terminal His_6_-tag into the pQE32 plasmid, generating plasmid pQE32-His_6_Q1ΔL and pQE32-His_6_Q1.

The Q2 chimera containing the Asp168Ala substitution was constructed by directed PCR mutagenesis using pQE32-His_6_Q1 plasmid as template. Fragment A spannings from the start codon of *aroK* to the codon that encodes the Asp36 residue was PCR-amplified with oligonucleotides 3′aroKquim (anneals at the 5′-end of the *aroK* start codon) and 3′D36A (5′-AATCTCTTGAGCGGAATCGTAAAATTC-3′; anneals at the region spanning codon Asp36 of the *aroK* gene and replaces it with an Ala codon). Fragment B, spanning from the codon that encodes the Asp36 residue to the 3′-end of *aroK,* was PCR-amplified with oligonucleotides 5'D36A (5′-GAATTTTACGATTCCGCTCAAGAGATT-3′; anneals at the region spanning codon Asp36 of the *aroK* gene and replaces it with an Ala codon) and 5'aroKquim (anneals at the 3′-end of *aroK*). A second round of PCR amplification uses as templates the A and B fragments, and as primers the 3'aroKquim and 5'aroKquim oligonucleotides, which anneal at the start and stop codon of the *aroK* gene, respectively. The resulting *aroK* mutant gene that contains the Asp36Ala substitution was *Kpn*I/*Pst*I-double digested, replacing the wild-type *aroK* gene cloned in pQE32-His_6_Q1 to give rise to plasmid pQE32-His_6_Q2.

All constructions were confirmed by DNA sequencing.

### Shikimate Kinase Activity Assays

Shikimate kinase activity was determined at 25°C by coupling the release of ADP to pyruvate kinase (EC 2.7.1.40) and lactate dehydrogenase (EC 1.1.1.27) reactions. Shikimate-dependent oxidation of NADH was monitored at 340 nm (ε = 6180 M^−1^ cm^−1^). The standard assay mixture contained (final concentration) 50 mM triethanolamine hydrochloride/KOH buffer, pH 7.0, 50 mM KCl, 5 mM MgCl_2_, 1.6 mM shikimate, 2.5 mM ATP, 1 mM phosphoenolpyruvate, 0.1 mM NADH, 3 units of pyruvate kinase (Sigma)/ml, 2.5 units of lactate dehydrogenase (Sigma)/ml, and the target protein to be assayed. *K_m_* values for shikimate were obtained from the Lineweaver-Burk plot [13]. Although it has been described that *E. coli* SKI has a high *K_m_* (5–20 mM) for shikimate, this value was obtained using an SKI isolated as a heterologous mix of oligomers [14]. Our results show that the His_6_-SKI has a *K_m_* for shikimate (0.4 mM) similar to that reported previously for the *E. coli* SKII [15]. In the competition assays, benzoyl-CoA or AMP were added at a 10 mM concentration to the standard assay conditions, and shikimate kinase activity was compared to that obtained in the absence of these inhibitors.

### Fluorescence Assays

Binding of benzoyl-CoA to CBzdR was determined by monitoring the change in protein fluorescence upon ligand addition. Measurements were obtained in an SML-Amicon 8000 spectrophotometer using quartz cuvettes and 0.2-cm excitation and 1-cm emission path lengths (λ_excitation_ = 275 nm and λ_emission_ 312 nm; slit widths = 5 nm). Titrations were performed at 25°C by adding a benzoyl-CoA stock solution to 0.4 ml of 20 mM Tris-HCl, pH 8.0, 100 mM KCl containing CBzdR (1 µM initial concentration). The final ligand concentration ranged from 0 to 300 µM. Corrections were made for protein dilutions, background signal, and for the instrument response as indicated by the manufacturer. Control titrations with buffer alone produced no change in the emission signal after correlation. The *K_d_* value for benzoyl-CoA in CBzdR was calculated by non-linear regression using the equation for a one-site saturation model ([*L*]*_b_*/[*P*]*_t_* = ([*L*]*_free_*/*K_d_*+[L]*_free_*) implemented in the GraphPad Prism software (GraphPad software, San Diego); where [*L*]*_b_*, is the concentration of bound ligand; [*P*]*_t_*, the total protein concentration, and [L]*_free_*, the concentration of free ligand. The fraction of bound ligand was calculated as (*I_o_*−*I*)/(*I_o_*−*I_min_*); where *I_o_* is the maximum CBzdR fluorescence intensity (no ligand added); *I* is the fluorescence intensity after addition of an aliquot of ligand, and *I_min_* is the fluorescence intensity at saturating concentration of ligand (this value was estimated by nonlinear regression to a single hyperbolic decay curve). The data obtained in at least three experimental trials were averaged to calculate the dissociation constant.

## Results and Discussion

### NBzdR Is a Functional DNA-Binding Domain

**Figure 2:**
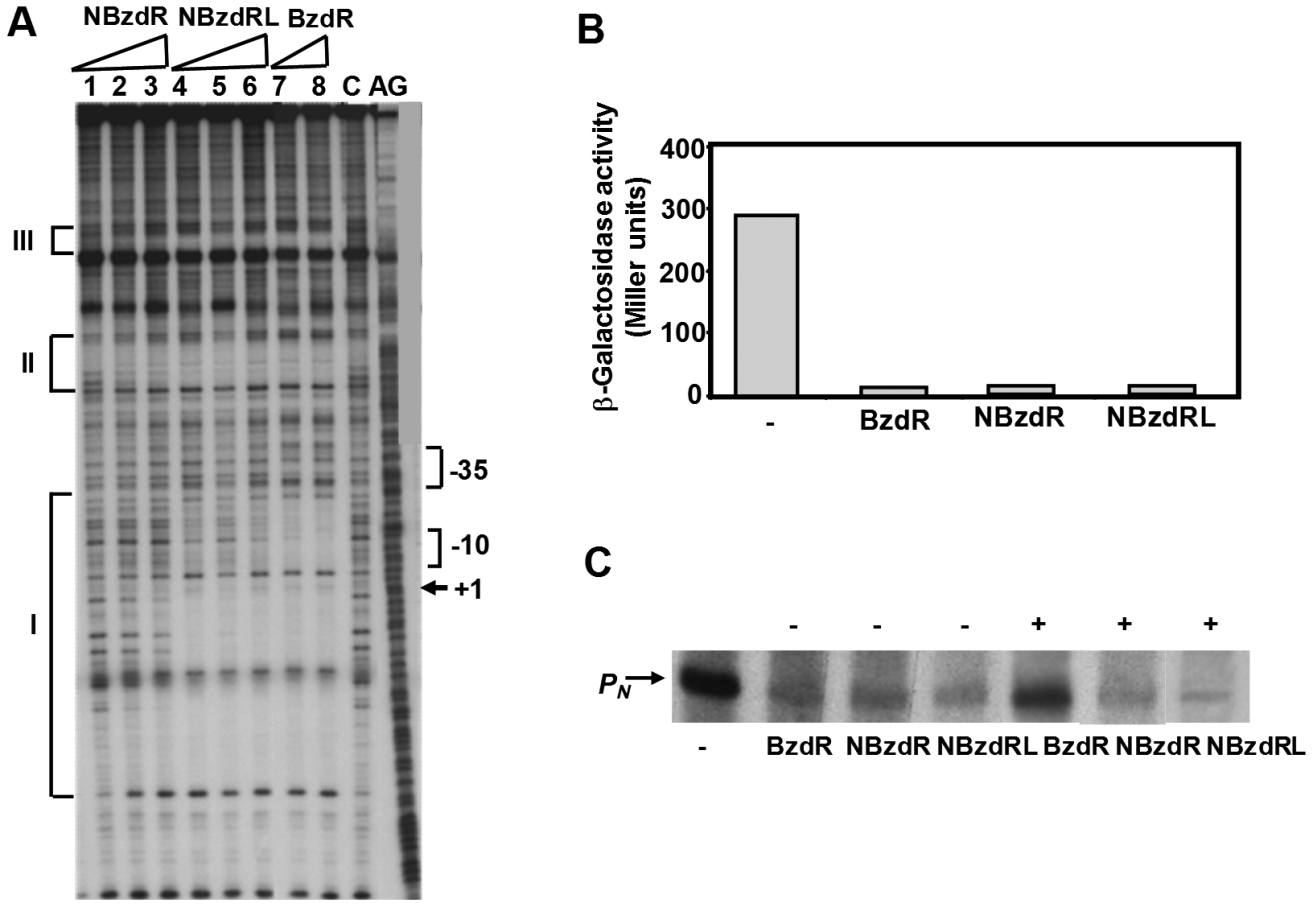
The N-terminal domain of BzdR is a functional DNA-binding domain. **A.** DNase I footprinting experiments were performed using the *P_N_* probe and the purified regulators His_6_-BzdR (control), His_6_-NBzdR, and His_6_-NBzdRL. The figure shows the results of footprinting assays conducted in the absence of the regulators (lane C), or the presence of 50, 100, or 200 nM of His_6_-NBzdR (lanes 1 to 3, respectively) or His_6_-NBzdRL (lanes 4 to 6, respectively). Lanes 7 and 8 are footprinting assays containing 50 and 100 nM of purified His_6_-BzdR. Lane AG shows the A+G Maxam and Gilbert sequencing reaction. Protected regions (I, II, and III) are indicated with brackets. The −10 box and the transcription initiation site (+1) of the *P_N_* promoter are also shown. Phosphodiester bonds hypersensitive to DNase I cleavage are indicated by asterisks. **B.** *In vivo* effect of the N-terminal domain of BzdR on the repression of the *P_N_* promoter. *E. coli* AFMCPN cells (containing a *P_N_::lacZ* fusion chromosome insertion of the) harboring plasmid pCK01BzdR (BzdR), pCK01NBzdR (NBzdR) or pCK01NBzdRL (NBzdRL) or the control plasmid pCK01 (-), were grown anaerobically in LB medium until the mid-exponential culture phase. β-galactosidase activity is expressed in Miller units. Results from three independent experiments (*n* = 3) and errors bars are shown. **C.** Effect of BzdR, NBzdR and NBzdRL on *in vitro* transcription from *P_N_*. Multiple-round *in vitro* transcription reactions were performed using the pJCD-P_N_ plasmid template, which produces a 184-nt mRNA from *P_N_* (arrow), 50 nM *E. coli* RNAP, and 20 nM Fnr* activator. Transcription reactions were carried out in the absence of repressor (lane -) or presence of 40 nM purified His_6_-BzdR (lanes BzdR), His_6_-NBzdR (lanes NBzdR) or His_6_-NBzdRL (lanes NBzdRL) and in the absence (-) or presence (+) of 2 mM benzoyl-CoA.

To confirm our hypothesis that the molecular architecture of BzdR arises from the fusion of two individual functional domains, the NBzdR and CBzdR domains were expressed, purified and characterized as independent proteins (Figure 1). Purified NBzdR protein was able to bind to the operator region of the *P_N_* promoter, as illustrated by gel shift (data not shown) and DNase I footprinting experiments (Figure 2A). The expression of NBzdR in *E. coli* cells with a chromosomal *P_N_::lacZ* fusion elicited the same repression of the *P_N_* promoter observed in cells expressing the entire BzdR regulator (Figure 2B). *In vitro* transcription experiments also revealed the ability of NBzdR to inhibit the *P_N_* promoter (Figure 2C). The predicted five α helices of NBzdR constitute the minimal structural domain since deletion of helix 5 (residues 77 to 86) did not allow for the production in *E. coli* of a stable protein that could be detected by SDS-PAGE (not shown).

We observed that when NBzdR was fused to the linker region, designated NBzdRL (residues 1 to 130; Figure 1), it was also able to repress the activity of *P_N_* both *in vivo* and *in vitro* (Figure 2B and Figure 2C). Moreover, NBzdRL showed greater protection to this promoter (operator regions I and II) than NBzdR and similarly to that observed for complete BzdR protein (Figure 2A). Gel shift experiments also revealed that NBzdRL was able to retard the migration of the *P_N_* promoter at lower concentrations than those required for NBzdR (data not shown). These results suggest that the BzdR linker sequence, whose main function is to facilitate interdomain communication [8], might also play a role in stabilization and binding of the N-terminal domain to the target DNA.

BzdR is a dimer in solution [8]. To test whether this property is conferred by the N-terminal domain, we examined the association state of the NBzdR and NBzdRL proteins. Sedimentation velocity experiments revealed that both proteins sedimented as single species with s-values of 1.8 and 2.3 S, respectively. Also, sedimentation equilibrium experiments confirmed that these species were dimers (Figure S1), demonstrating that BzdR dimerization is an intrinsic property of the N-terminal domain.

We should mention that benzoyl-CoA was unable to rescue the repression caused by NBzdR or NBzdRL over the *P_N_* promoter both *in vitro* (Figure 2C) and *in vivo* (data not shown), confirming that NBzdR is not the target of the inducer molecule and, therefore, behaves as a super-repressor. All of these results suggest that NBzdR is a true functional domain that retains the ability of native BzdR protein to bind the *P_N_* promoter and repress its activity.

### CBzdR Is a Functional Benzoyl-CoA-Binding Domain

The modelled 3D structure of CBzdR suggests the presence of a deep groove that could interact with the benzoyl-CoA inducer molecule [7], triggering a conformational change in BzdR protein [8]. To establish if effector binding was specific of the C-terminal domain of BzdR, we investigated whether CBzdR would interact with benzoyl-CoA. As expected, fluorescence emission by CBzdR was dramatically modified upon incubation with benzoyl-CoA (Figure S2). Based on the structural model for CBzdR [7], fluorimetry data could be fitted to a single binding site, indicating the formation of a weakly bound CBzdR-benzoyl-CoA complex (*K*_d_ 203±35 µM) as previously observed for the parent BzdR regulator (*K*_d_ 157±58 µM) [8]. This suggests that benzoyl-CoA induces a conformational change in CBzdR and that it therefore, retains its effector-binding ability in the absence of the NBzdR domain, thus behaving as an independent functional domain.

Analytical ultracentrifugation experiments revealed that CBzdR behaves as a single species with an s-value of 1.8 S, compatible with a globular monomer (Figure S3). The observation that CBzdR is a monomer that undergoes conformational changes in the presence of its cognate ligand (benzoyl-CoA), resembles the behaviour of SKs which undergo extensive conformational changes in the presence of their substrates, i.e., ATP and shikimate [8]. However, despite this conserved similarity with SKs, CBzdR showed no detectable shikimate kinase activity (Figure S4A).

### The Q1 Chimera: a Bifunctional Regulator that Controls the *P_N_* Promoter *in vitro*

The results described above demonstrate that the organization of BzdR is modular and shaped by two functional domains, i.e., NBzdR, which binds to the target promoter and CBzdR, which recognizes benzoyl-CoA as its inducer molecule. This molecular architecture suggests that BzdR may have arisen from the fusion of an HTH-XRE DNA-binding domain to a protein structurally resembling SK or an ancestor of this enzyme. The structural similarity between ATP and shikimate, the two substrates of SK, and the two ends of the benzoyl-CoA molecule, i.e., 3P-ADP and the benzoyl group, suggest that the latter could also be recognized by the SK enzyme. In agreement with this hypothesis, we previously observed that benzoyl-CoA inhibits (by 44%) *E. coli* SKI activity in a similar manner to AMP (34% inhibition), a well-known SK inhibitor [11], suggesting that benzoyl-CoA can be considered a newly identified ligand of the well-studied SK enzyme.

To unravel the evolutionary pathway that could have given rise to the current BzdR regulator, we used a protein engineering approach and constructed the chimeric protein Q1 by the fusing NBzdRL to *E. coli* SKI protein (Figure 1). Interestingly, Q1 showed SK activity yielding a *K*_m_ for shikimate of 0.6 mM, similar to that exhibited by *E. coli* SKI purified at our laboratory (*K*_m_ 0.4 mM) (Figure S4A). The enzyme activity of the SK domain in the Q1 chimera was also demonstrated *in vivo* in an *E. coli aroK aroL* double mutant strain lacking SK activity that is unable to grow in minimal medium since it cannot synthesize aromatic amino acids [11]. *E. coli aroK aroL* cells transformed with a plasmid that expressed the Q1 protein were observed to grow in glycerol-containing minimal medium (Figure S4B), indicating that Q1 was able to rescue the lack of SK activity in the mutant strain.

In analytical ultracentrifugation experiments, Q1 protein behaved as a single species with an s-value of 3.7 S consistent with a protein dimer (Figure S3). This finding is in agreement with the fact that this chimeric protein contains the NBzdR homodimeric domain, and reveals that dimerization does not prevent the catalytic activity of the monomeric SKI enzyme.

**Figure 3:**
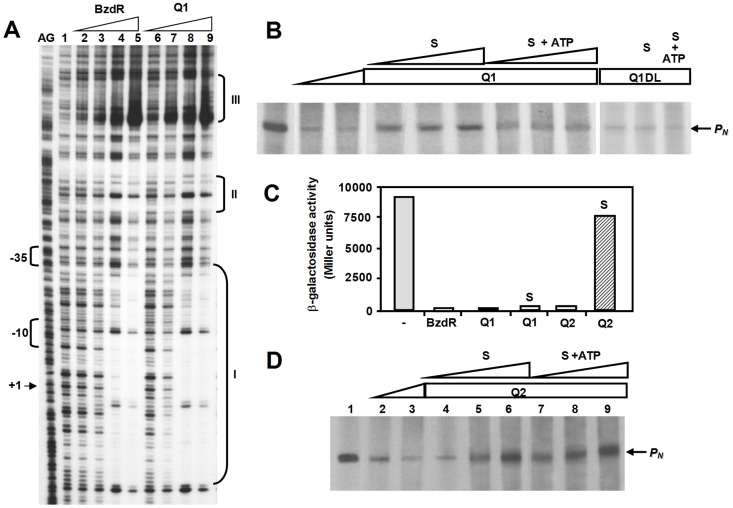
*In vitro* and *in vivo* effects of the Q1, Q1ΔL and Q2 chimeras on the *P_N_* promoter. (**A**) DNase I footprinting experiments performed out using the *P_N_* probe and the purified regulators His_6_-BzdR (control) and His_6_-Q1. The figure shows the results of footprinting assays conducted in the absence of the regulators (lane C), or presence of 25, 50, 100, and 200 nM of His_6_-BzdR (lanes 1 to 4, respectively) or His_6_-Q1 (lanes 5 to 8, respectively). Lane AG shows the A+G Maxam and Gilbert sequencing reaction. Protected regions (I, II, and III) are indicated by brackets. The -10 box and the transcription initiation site (+1) of the *P_N_* promoter are also shown. Phosphodiester bonds hypersensitive to DNase I cleavage are indicated by asterisks. (**B**) *In vitro* effect of the Q1 and Q1ΔL chimeras on the activity of *P_N_*. Multiple-round *in vitro* transcription reactions were performed using the pJCD-P_N_ plasmid template, which produces a 184-nt mRNA from *P_N_* (arrow), 50 nM *E. coli* RNA polymerase (RNAP), and 20 nM Fnr* activator [12]. Transcription reactions were conducted in the absence of chimeric regulator (lane 1) or presence of 25 nM (lane 2) or 50 nM (lanes 3–12) of purified His_6_-Q1 or His_6_-Q1ΔL proteins. Shikimate (S) or shikimate plus ATP (S+ATP) were added at 1 mM (lanes 4 and 7), 2 mM (lanes 5 and 8) or 4 mM (lanes 6, 9, 11 and 12). (**C**) *In vivo* effect of BzdR (control) and the Q1 and Q2 chimeras on the activity of the *P_N_* promoter. β-galactosidase activity (in Miller units) of *E. coli* MC4100 cells harboring plasmid pSJ3P_N_ (*P_N_::lacZ*) and the plasmids pCK01BzdR (BzdR), pCK01Q1 (Q1), pCK01Q2 (Q2), or the control plasmid pCK01 (-). Cells were grown anaerobically until mid-exponential phase in LB medium supplemented, when indicated, with 5 mM shikimate (S). Results from three independent experiments (*n* = 3) and errors bars are shown. (**D**). *In vitro* effect of the Q2 chimera on the activity of *P_N_*. *In vitro* transcription reactions were performed as in *panel B*, in the absence of Q2 (lane 1) or presence of 25 nM (lane 2) or 50 nM (lanes 3–9) of purified His_6_-Q2 protein. Shikimate (S) or shikimate plus ATP (S+ATP) were added at 1 mM (lanes 4 and 7), 2 mM (lanes 5 and 8) or 4 mM (lanes 6 and 9).

The Q1 chimera was able to bind to the three operator regions of the *P_N_* promoter conferring similar protection to that shown by the BzdR regulator (Figure 3A). In addition, it was capable of inhibiting *P_N_* promoter activity *in vitro* (Figure 3B, lanes 2-3). Accordingly, we can conclude that both domains are fully and separately active in the Q1 chimera.

**Figure 4:**
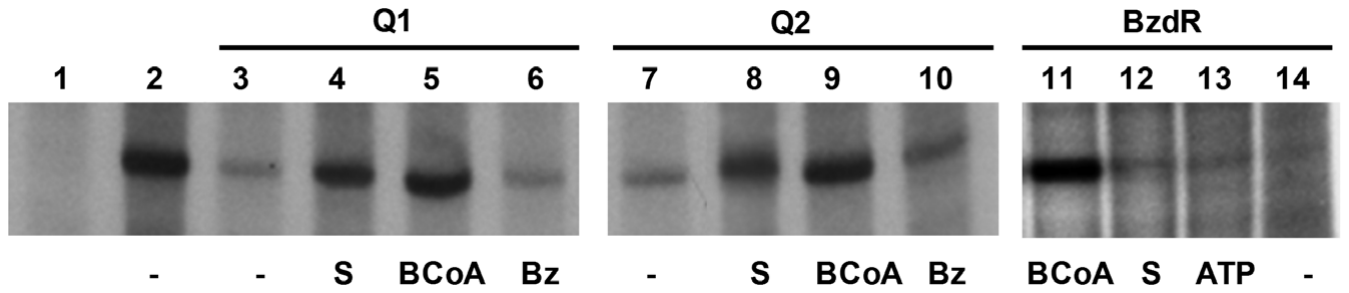
Effect of different ligands on BzdR-, Q1- or Q2-mediated repression of *P_N_*. *In vitro* transcription reactions were run as in Figure 2B, in the absence of repressor proteins (lane 2) or presence of 40 nM purified His_6_-Q1 (lanes 3 to 6), His_6_-Q2 (lanes 7 to 10) or His_6_-BzdR (lanes 11 to 14). The ligands shikimate (S) (lanes 4, 8, 12), benzoyl-CoA (BCoA) (lanes 5, 9, 11), and benzoate (Bz) (lanes 6, 10) were added at 1 mM; ATP was added at 4 mM (lane 13). Lanes -, no ligand added. Lane 1, control assay without RNAP.

It is well known that SK enzymes undergo profound conformation changes when they interact with their substrates, ATP-Mg^2+^ and shikimate. The binding of shikimate leads to a structural state (loaded conformation) that, in the presence of ATP-Mg^2+^, is maintained for a short period of time sufficient to trigger the catalytic reaction to produce 3P-shikimate. The enzyme subsequently recovers its starting structural state (unloaded conformation) (Figure S5) [16], [17]. Since the Q1 chimera is able to bind and inhibit the activity of the *P_N_* promoter in the absence of shikimate and ATP (Figures 3A and 3B), we anticipated that the presence of shikimate alone could provoke a conformational change in the SK domain, i.e., to a loaded-like conformation, that would lead to the release of the Q1 protein from the *P_N_* promoter. When increasing concentrations of shikimate were added to the in vitro transcription assay mix, significant *P_N_* promoter de-repression was observed (Figure 3B, lanes 4–6). Moreover, this de-repression was not observed when the Q1 chimera was replaced with Q1ΔL protein (Figure 3B, lanes 10–12), a new chimera lacking the linker region between the NBzdR and SK domains (Figure 1). These data suggest that the Q1 chimera is a bifunctional regulator that controls *P_N_* promoter activity and responds to shikimate as an inducer molecule *in vitro* by generating a conformational change transmitted to the DNA-binding domain through the linker region. Interestingly, benzoyl-CoA was also shown to offset the Q1-dependent repression of *P_N_* (Figure 4, lane 5) with an efficiency similar to that shown by shikimate (Figure 4, lane 4) but lower than that observed for the native BzdR (Figure 4, lane 11). As previously noted for BzdR [7], benzoate could not mimic the inducer effect of its CoA derivative (benzoyl-CoA) in the Q1 protein (Figure 4, lane 6), emphasizing the importance of the CoA moiety in effector recognition.

The observation that CBzdR lacks SK activity (Figure S4A) raises the question of whether the existence of such activity in the Q1 chimera may interfere *in vivo* with effector-dependent de-repression of the *P_N_* promoter. This possibility was confirmed by monitoring *P_N_* promoter activity in *E. coli* cells expressing the *P_N_*::*lacZ* reporter fusion and the bifunctional Q1 chimera. It was observed that Q1 protein inhibited the activity of *P_N_* as efficiently as the control BzdR repressor even after the addition of shikimate to the culture medium (Figure 3C). These data strongly suggest that the presence in the cell of both substrates of SK, i.e., shikimate and ATP-Mg^2+^, prevents de-repression of the *P_N_* promoter. The lack of *P_N_* induction by shikimate *in vivo* was also confirmed *in vitro* when ATP-Mg^2+^ and shikimate were added together to the transcription assay (Figure 3B, lanes 7–9). These results are in agreement with the rapid conformational changes observed in the SK enzyme after binding of shikimate and ATP-Mg^2+^ during catalysis [18], [19], and suggest the SK domain of the Q1 protein does not remain in the loaded-like conformation (Figure S5) for sufficient time to trigger the release of the repressor from the target promoter.

Taken together, these results confirm that the SK activity of the Q1 regulator prevents effector-specific de-repression of the target promoter by shikimate under physiological conditions.

### Evolving the Q1 Chimera towards a BzdR-like Regulator: the Q2 Chimera

The results reported above suggest that CBzdR may have evolved from an SK-like enzyme that lost its enzyme activity while maintaining its benzoyl-CoA binding ability to efficiently control the *P_N_* promoter. To simulate the predicted molecular history of BzdR in the laboratory, we engineered a Q1 derived protein containing a single mutation that abolished its enzyme activity.

Since SK activity is strictly dependent on Mg^2+^, and binding of this metal requires one Thr and two Asp conserved residues [19], we engineered a new synthetic protein, a so-called Q2 chimera (Figure 1), by replacing the Asp168 residue of the Q1 protein (corresponding to Asp36 of SKI) with an Ala residue. This substitution avoids Mg^2+^ binding while maintaining the overall 3D structure of the SK domain. As expected, the purified Q2 protein showed no SK activity (Figure S4A), but was still able to efficiently inhibit the *in vitro* activity of the *P_N_* promoter (Figure 3D, lanes 2–3). The addition of shikimate or benzoyl-CoA (but not benzoate) to the *in vitro* transcription reaction released the repression of the *P_N_* promoter (Figure 3D, lanes 4–6; Figure 4, lanes 8–10) suggesting that the SK domain of Q2 retained its ability to bind both ligands and transfer the corresponding conformational changes to the NBzdR domain. Interestingly, shikimate also attenuated the repression exerted by Q2 even in the presence of ATP-Mg^2+^ (Figure 3D, lanes 7–9). Moreover, *in vivo* experiments revealed that the addition of shikimate to the culture medium of *E. coli* cells expressing the *P_N_*::*lacZ* reporter fusion and the Q2 chimera reduced the repression exerted by Q2 (Figure 3C), a behaviour not observed for the Q1 chimera. These results indicate that when the *P_N_* promoter is repressed by the Q2 chimera it can be de-repressed *in vivo* by shikimate.This observation is consistent with the fact that the catalytically inactive SK domain of the synthetic Q2 regulator can adopt a permanent loaded-like conformation under physiological conditions when shikimate is present, thus triggering the release of the repressor from the target promoter.

All these results point to a role of the Q2 chimera as a synthetic transcriptional regulator that can control the *P_N_* promoter by recognizing shikimate and benzoyl-CoA as inducer molecules. Shikimate and ATP were not effective CBzdR ligands since they were unable to significantly impair the BzdR-dependent inhibition of *P_N_* (Figure 4, lanes 12–13). Thus, the Q2 chimeric protein represents a BzdR-like regulator with a broader effector-binding range and is a likely intermediate in the evolutionary history of the current BzdR protein.

Several lines of research have suggested that early enzyme forms exhibited broad substrate specificity, or “substrate ambiguity”, and later diverged to give rise to the specific enzymes we know today [20], [21], [22], [23]. Proteins acquire new functions without losing their original function, and gene duplication may follow the emergence of a new function, rather than initiate it [24]. In effect, it is known that the SK domain can occur as an independent enzyme, e.g., AroK and AroL proteins in bacteria [15], or as a domain of multifunctional enzymes, e.g., AROM protein in Ascomycetes [25]. Moreover, the duplication of genes coding for SK-like domains is very common in plants and some of these proteins have acquired novel functions, e.g., SKL1, which seems essential for chloroplast biogenesis and SKL2, which encodes a key protein for species-adaptive molecular evolution in *Arabidopsis thaliana* [26]. Although these duplications are less frequent in prokaryotes, *E. coli* also has two SKs, and one of these (SKI) could play a role in an as yet unknown cell function since *aroK* mutants are resistant to mecillinam [27]. In contrast with examples proposed previously whereby an ancestral SK evolved into a novel enzyme, our work shows that the SK domain could also have evolved to give rise to a regulatory protein. We suggest that SK behaves as a “stem” protein domain, able to interact with different substrates and thus facilitating the acquisition of new functions over time. In effect, a similar SK domain has also been detected in a putative regulator (plpp0115 gene product) in the γ-proteobacterium *Legionella pneumophila* [28].

### Concluding Remarks

**Figure 5:**
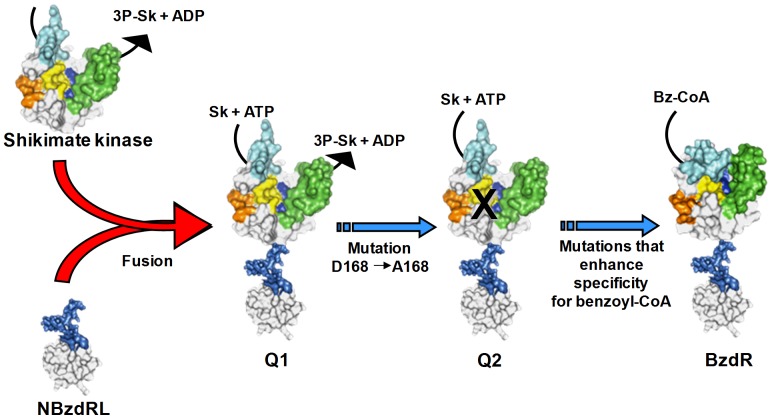
Proposed model for the evolutionary origin of the BzdR protein. The red arrows represent fusion between a shikimate kinase ancestor and a DNA-binding domain of the HTH-XRE family. This fusion rendered a protein equivalent to the Q1 chimera. A series of mutations led to the loss of enzyme activity (chimera Q1) and finally to increase in the affinity of the protein for the inducer benzoyl-CoA. Sk: shikimate; 3-P-Sk: 3-phosphoshikimate; Bz-CoA: benzoyl-CoA.

We report the reconstruction of an analogue of an ancestral protein that could have generated one of the domains of the current BzdR regulator, whose modular architecture comprises two independent functional domains, i.e., a DNA-binding domain (NBzdR) and an effector-binding domain (CBzdR) (Figure 5). The fact that we were able to construct and characterize a bifunctional Q1 regulator strongly suggests that fusion of an HTH-XRE DNA-binding domain to an SK domain was a major step in the evolution of BzdR. In addition, this SK domain was found to behaves as a stem protein domain able to interact with different substrates. The finding that SK recognizes benzoyl-CoA provides additional evidence for our hypothesis. However, the loss of enzyme activity of the SK domain may have been a critical event to improve the efficiency of the ancestral BzdR regulatory protein. Indeed, this was indicated by the catalytically-inactive Q2 chimera which, unlike Q1, was able to respond *in vivo* to the inducer molecules. This, synthetic Q2 protein constitutes the first shikimate-dependent transcriptional regulator described so far. Our finding that it is possible to construct a transcriptional regulator by fusing an appropriate DNA-binding domain to an enzyme that recognizes the target compound, paves the way for a novel rational approach to create new *à la carte* regulatory proteins. During the course of evolution of the BzdR regulator, the Q2-like ancestral protein proposed likely suffered additional mutations preventing its recognition of shikimate while increasing its binding affinity for benzoyl-CoA, the inducer and first intermediate of the catabolic pathway controlled by BzdR.

Besides recreating a plausible pathway for the evolution of an enzyme into a transcriptional regulator, our findings also support the notion that enzymes preceded the emergence of the regulatory systems that control their expression.

## Supporting Information

Figure S1 **Study of the oligomerization state of NBzdR (A) and NBzdRL (B) proteins in solution.** Sedimentation equilibrium data (grey dots) and best fit analysis assuming a protein dimer (black line) and monomer (red line) species. The lower panels show the difference between estimated values and experimental data for protein dimers (residuals). The data indicate that NBzdR and NBzdRL proteins are dimers, demonstrating that BzdR dimerization is an intrinsic property of the N-terminal domain.
(TIF)

Figure S2 **Conformational changes in CBzdR induced by benzoyl-CoA binding.** Intrinsic fluorescence of His_6_-CBzdR as a function of benzoyl-CoA (Bz-CoA) concentration. Data points represent the decrease in the His_6_-CBzdR (7.5 µM) fluorescence emission maximum (312 nm) expressed in arbitrary units (a.u.) upon excitation at 275 nm in the presence of increasing concentrations of Bz-CoA. Insets, fitting Bz-CoA binding to His_6_-CBzdR [P] to a single site model. This result suggests that benzoyl-CoA induces a conformational change in CBzdR and, therefore, that CBzdR retains its effector-binding ability in the absence of the NBzdR domain, thus behaving as an independent functional domain.
(TIF)

Figure S3 **Study of the oligomerization state of CBzdR and Q1 proteins in solution.** Sedimentation coefficient distribution c(s) corresponding to the sedimentation velocity of purified His_6_-CBzdR (broken blue line) and His_6_-Q1 (solid blue line) proteins. The protein concentration distribution pattern (c(s)) and sedimentation coefficient (S) are represented in the graph. The standard s-value of the protein did not change significantly with protein concentration over the range examined (1-30 µM). The Q1 protein behaved as a single species with an s-value 3.7 S consistent with a protein dimer, whereas CBzdR behaved as a single species with an s-value of 1.8 S compatible with a globular monomer.
(TIF)

Figure S4 **Shikimate kinase activity of** ***E. coli*** **SKI and CBzdR, Q1 and Q2 proteins.** **A.** SK activities are shown for the purified His_6_-SKI (red line), His_6_-CBzdR (orange line), His_6_-Q1 (black line) and His_6_-Q2 (green line). Despite its conserved similarity with SKs, CBzdR showed no detectable shikimate kinase activity. **B.** Growth curves for *E. coli* ALO807strain defective in the *aroL* and *aroK* genes harboring plasmid pQE32-His_6_Q1 (expresses the His_6_-Q1 protein) (red lines) or the control pQE32 plasmid (green lines). Cells were grown in M63 minimal medium supplemented with 30 mM glycerol in the presence (continuous lines) or absence (discontinuous lines) of 0.4% (w/v) casamino acids. The results of a single representative experiment are shown, and values were reproducible in three separate experiments with standard deviation values of <10%. These results demonstrate that the Q1 protein retains SK activity in the SK defective mutant strain.
(TIF)

Figure S5 **Diagram showing the conformational changes the SK enzyme undergoes during a catalytic cycle.** The SK enzyme shows the LID region (white), the NB region (green) responsible for ATP-recognition, the ESB region (blue) involved in shikimate recognition, and the RC region (red), which constitutes the rest of the SK structure. **A.** “Unloaded conformation” of the SK enzyme. **B.** “Loaded conformation” of SK in the presence of shikimate. **c.** Structural conformation of the SK in the presence of ATP-Mg^2+^ and shikimate. Thick black arrow represents the release of the two products of the reaction, 3P-shikimate and ADP, and the recovery of the initial “unloaded conformation” of the SK enzyme. Adapted from Hatmann *et al*. [17]
(TIF)
