# Supplementary figures and images for: Identification of a Missing Link in the Evolution of an Enzyme into a Transcriptional Regulator
Source: PLoS One. 2013 Mar 19;8(3):e57518. doi: 10.1371/journal.pone.0057518 (PMC3602430; doi:10.1371/journal.pone.0057518)

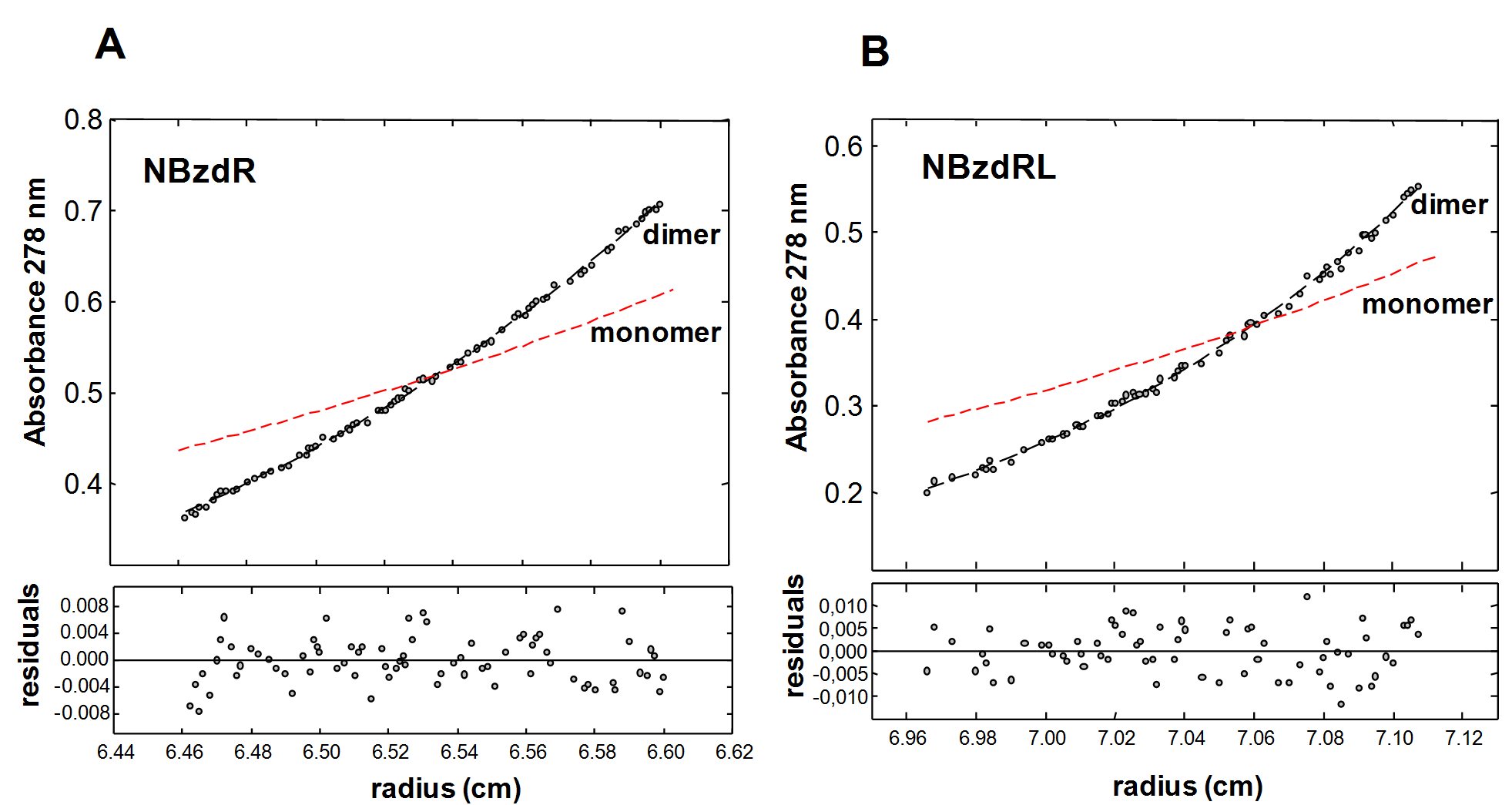

Supplement: Figure S1 — Study of the oligomerization state of NBzdR (A) and NBzdRL (B) proteins in solution. Sedimentation equilibrium data (grey dots) and best fit analysis assuming a protein dimer (black line) and monomer (red line) species. The lower panels show the difference between estimated values and experimental data for protein dimers (residuals). The data indicate that NBzdR and NBzdRL proteins are dimers, demonstrating that BzdR dimerization is an intrinsic property of the N-terminal domain. (TIF) [file pone.0057518.s001.tif]

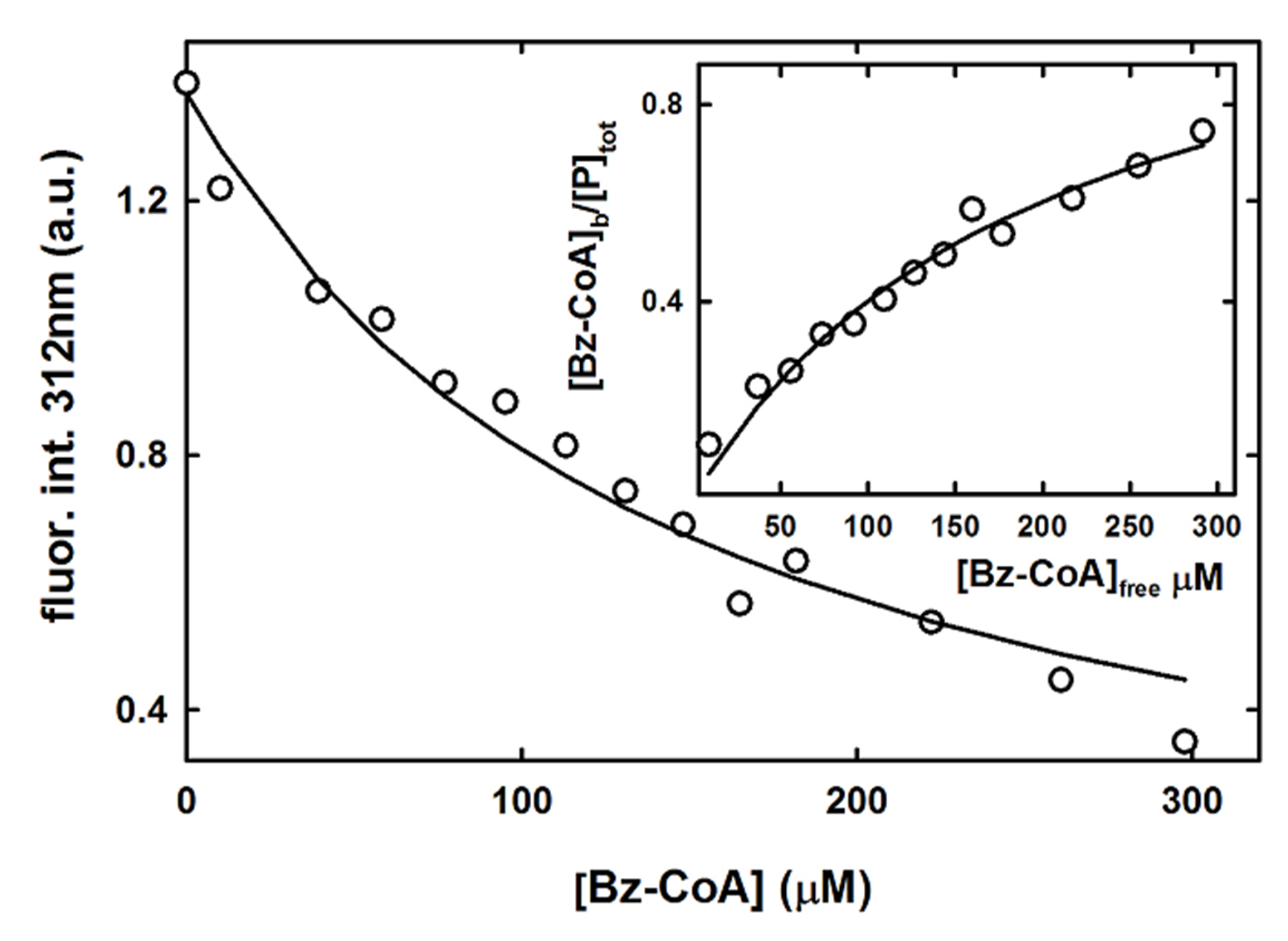

Supplement: Figure S2 — Conformational changes in CBzdR induced by benzoyl-CoA binding. Intrinsic fluorescence of His6-CBzdR as a function of benzoyl-CoA (Bz-CoA) concentration. Data points represent the decrease in the His6-CBzdR (7.5 µM) fluorescence emission maximum (312 nm) expressed in arbitrary units (a.u.) upon excitation at 275 nm in the presence of increasing concentrations of Bz-CoA. Insets, fitting Bz-CoA binding to His6-CBzdR [P] to a single site model. This result suggests that benzoyl-CoA induces a conformational change in CBzdR and, therefore, that CBzdR retains its effector-binding ability in the absence of the NBzdR domain, thus behaving as an independent functional domain. (TIF) [file pone.0057518.s002.tif]

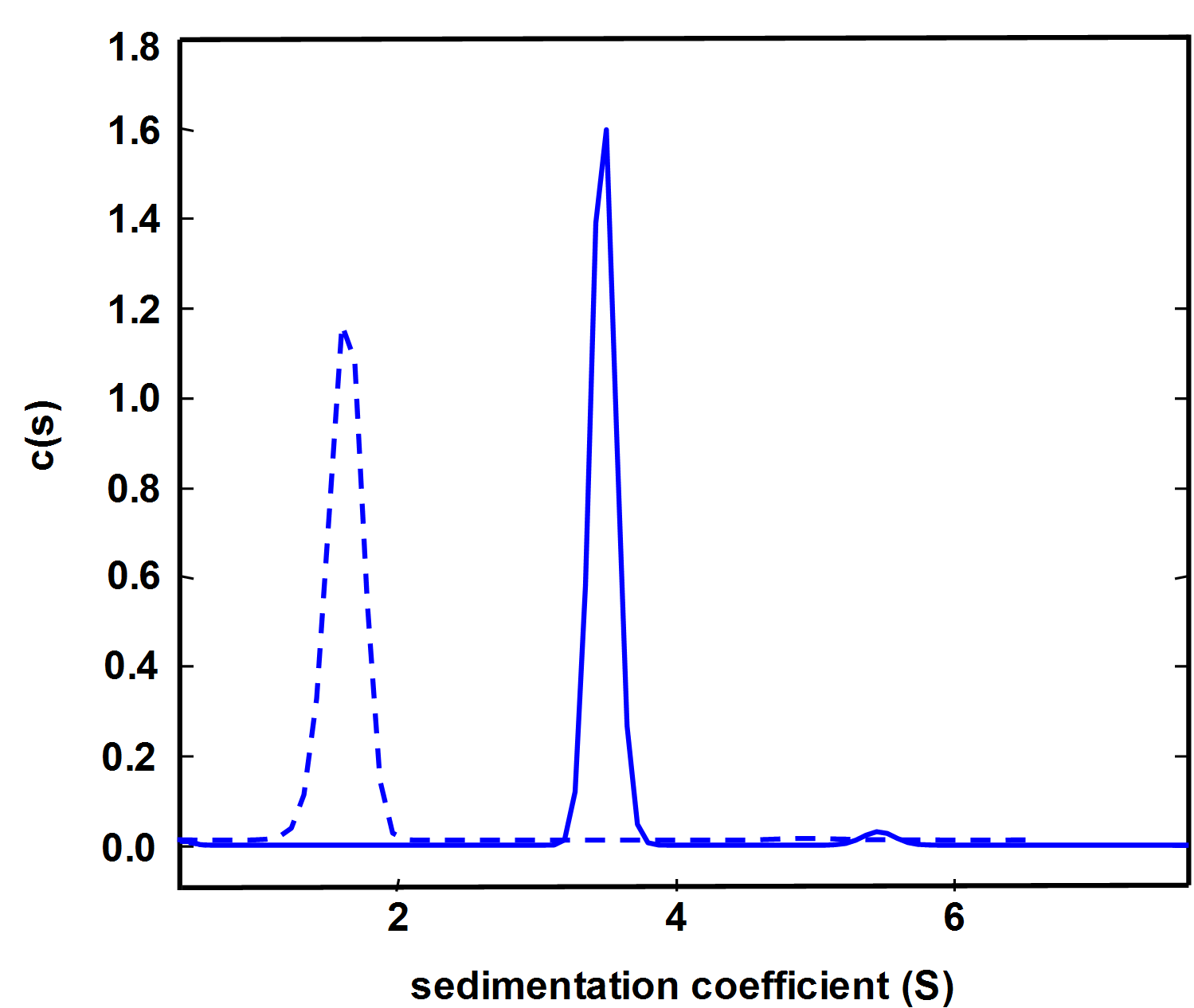

Supplement: Figure S3 — Study of the oligomerization state of CBzdR and Q1 proteins in solution. Sedimentation coefficient distribution c(s) corresponding to the sedimentation velocity of purified His6-CBzdR (broken blue line) and His6-Q1 (solid blue line) proteins. The protein concentration distribution pattern (c(s)) and sedimentation coefficient (S) are represented in the graph. The standard s-value of the protein did not change significantly with protein concentration over the range examined (1-30 µM). The Q1 protein behaved as a single species with an s-value 3.7 S consistent with a protein dimer, whereas CBzdR behaved as a single species with an s-value of 1.8 S compatible with a globular monomer. (TIF) [file pone.0057518.s003.tif]

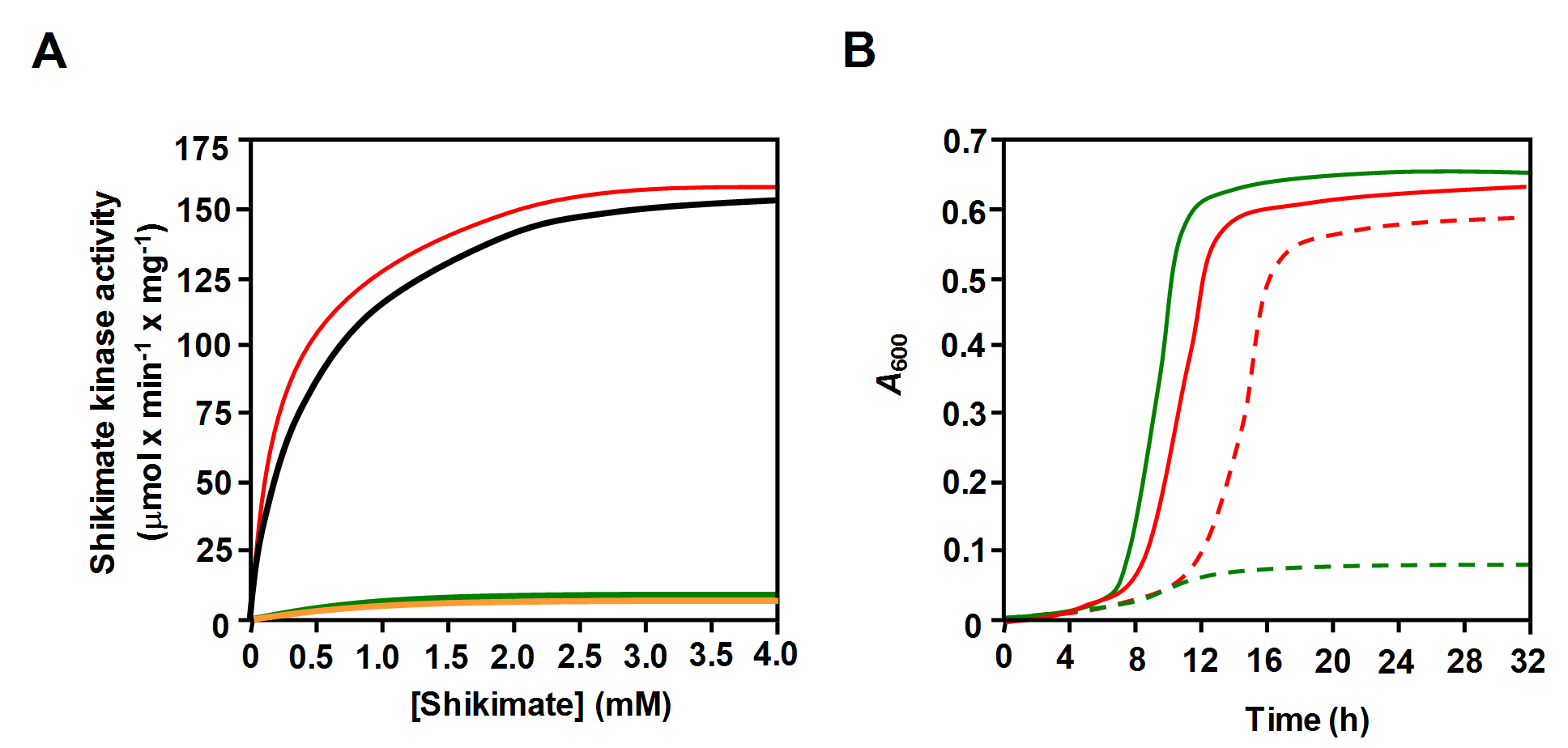

Supplement: Figure S4 — Shikimate kinase activity of E. coli SKI and CBzdR, Q1 and Q2 proteins. A. SK activities are shown for the purified His6-SKI (red line), His6-CBzdR (orange line), His6-Q1 (black line) and His6-Q2 (green line). Despite its conserved similarity with SKs, CBzdR showed no detectable shikimate kinase activity. B. Growth curves for E. coli ALO807strain defective in the aroL and aroK genes harboring plasmid pQE32-His6Q1 (expresses the His6-Q1 protein) (red lines) or the control pQE32 plasmid (green lines). Cells were grown in M63 minimal medium supplemented with 30 mM glycerol in the presence (continuous lines) or absence (discontinuous lines) of 0.4% (w/v) casamino acids. The results of a single representative experiment are shown, and values were reproducible in three separate experiments with standard deviation values of <10%. These results demonstrate that the Q1 protein retains SK activity in the SK defective mutant strain. (TIF) [file pone.0057518.s004.tif]

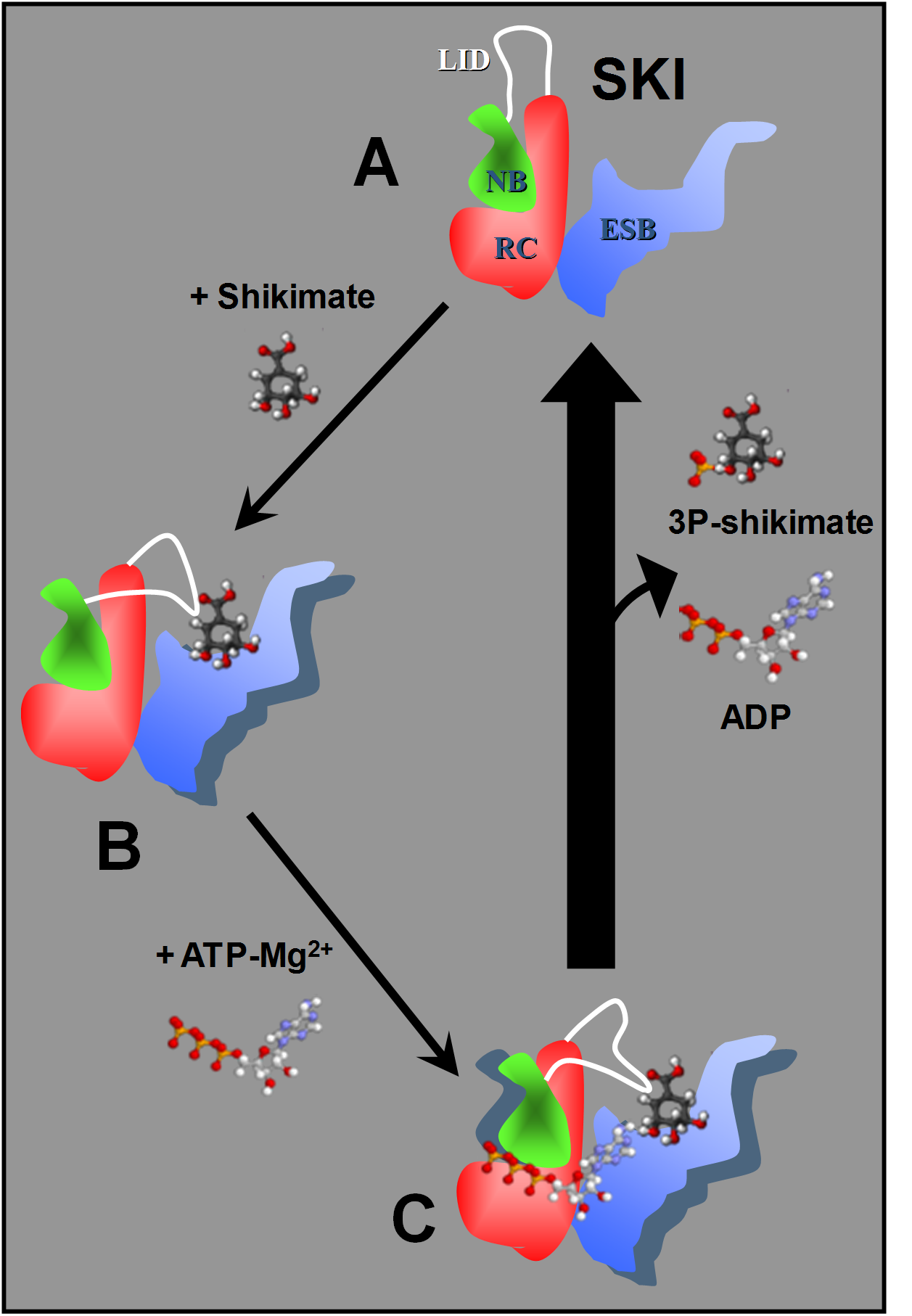

Supplement: Figure S5 — Diagram showing the conformational changes the SK enzyme undergoes during a catalytic cycle. The SK enzyme shows the LID region (white), the NB region (green) responsible for ATP-recognition, the ESB region (blue) involved in shikimate recognition, and the RC region (red), which constitutes the rest of the SK structure. A. “Unloaded conformation” of the SK enzyme. B. “Loaded conformation” of SK in the presence of shikimate. c. Structural conformation of the SK in the presence of ATP-Mg2+ and shikimate. Thick black arrow represents the release of the two products of the reaction, 3P-shikimate and ADP, and the recovery of the initial “unloaded conformation” of the SK enzyme. Adapted from Hatmann et al. [17] (TIF) [file pone.0057518.s005.tif]
